# Zebrafish Models for Skeletal and Extraskeletal Osteogenesis Imperfecta Features: Unveiling Pathophysiology and Paving the Way for Drug Discovery

**DOI:** 10.1007/s00223-024-01282-5

**Published:** 2024-09-25

**Authors:** Cecilia Masiero, Carla Aresi, Antonella Forlino, Francesca Tonelli

**Affiliations:** Department of Molecular Medicine, Biochemistry Unit, University of Pavia, Via Taramelli 3B, 27100 Pavia, Italy

**Keywords:** Zebrafish, Osteogenesis imperfecta, Bone, Extraskeletal tissues, Drug discovery

## Abstract

In the last decades, the easy genetic manipulation, the external fertilization, the high percentage of homology with human genes and the reduced husbandry costs compared to rodents, made zebrafish a valid model for studying human diseases and for developing new therapeutical strategies. Since zebrafish shares with mammals the same bone cells and ossification types, it became widely used to dissect mechanisms and possible new therapeutic approaches in the field of common and rare bone diseases, such as osteoporosis and osteogenesis imperfecta (OI), respectively. OI is a heritable skeletal disorder caused by defects in gene encoding collagen I or proteins/enzymes necessary for collagen I synthesis and secretion. Nevertheless, OI patients can be also characterized by extraskeletal manifestations such as dentinogenesis imperfecta, muscle weakness, cardiac valve and pulmonary abnormalities and skin laxity. In this review, we provide an overview of the available zebrafish models for both dominant and recessive forms of OI. An updated description of all the main similarities and differences between zebrafish and mammal skeleton, muscle, heart and skin, will be also discussed. Finally, a list of high- and low-throughput techniques available to exploit both larvae and adult OI zebrafish models as unique tools for the discovery of new therapeutic approaches will be presented.

## Introduction

Osteogenesis imperfecta (OI) is a family of inherited bone dysplasias characterized by a wide range of genetic and phenotypic variability [1]. Commonly referred to as “brittle bone disease”, OI is a rare collagen I-related condition affecting approximately 1 in 15–20 000 births and patients are mainly characterized by reduced bone mineral density (BMD), increased bone fragility, increase susceptibility to fractures, presence of dentinogenesis imperfecta and in several cases by extraskeletal features such as blue sclerae, hearing loss, ligamentous laxity, pulmonary disfunctions, cardiac valve abnormalities and muscle weakness [2–4]. Traditionally considered an autosomal dominant disorder, OI is primarily caused by dominant mutations in *COL1A1* and *COL1A2* genes, affecting collagen I structure. Nevertheless, over the past 20 years, many other OI forms have been identified predominantly associated to recessive and X-linked mutations affecting several genes encoding proteins and enzymes involved in collagen I synthesis, osteoblast differentiation and function [5].

The deep investigation of the disease pathophysiology can take advantage of several OI animal models either carrying spontaneous mutations or defects introduced by genetic manipulation. Naturally occurring OI has been described in Charolais cattle, sheep, cats and dogs [6–12]. However, only in few cases the molecular defect has been characterized and biochemical studies performed [11, 13, 14]. The most frequently used models to study OI are rodents due to the possibility to introduce knock out or knock in defects in their genome associated to their pathophysiological similarities to humans [15].

In the last decades *Danio rerio* (*D. rerio*) also emerged as a new powerful vertebrate model to dissect the molecular mechanisms of OI and as powerful tool for drug discovery [16]. Indeed, the external development, the embryo and larvae transparency, the impressive number of embryos from a single mating, the early skeletal formation from 4–5 days post fertilization (dpf) and the possibility to deliver drugs in water made zebrafish a unique tool to investigate bone in health and disease [17].

Collagen I in zebrafish is ubiquitously expressed as in mammals, anyway it should be considered that it is an heterotrimer constituted by the folding of three and not two different α chains. Indeed, as a result of the fish-specific whole genome duplication [18], zebrafish has two orthologous genes of human *COL1A1* namely *col1a1a* and *col1a1b*, which encode the α1 and the α3 chains, respectively. The α1(I) and α3(I) chains assemble with the α2(I) encoded by *col1a2* orthologue of human *COL1A2*. *col1a1a*, *col1a1b* share similar spatial–temporal expression pattern starting from the zygote stage until adulthood and the α1 and α3 chains have the same electrophoretic migration and similar amino acid composition, confirming the origin from a common ancestor. The collagen I C-propeptide domain fundamental for proα(I) chain recognition as well as the collagen-non collagenous protein binding domains are also highly conserved between human and zebrafish [19].

Concerning the skeleton, zebrafish shares with mammals the bone cell types, their gene expression profiles and the ossification processes [20, 21]. Furthermore, zebrafish bones undergo remodeling mediated by the activity of osteoblasts and osteoclasts miming mammals’ bone turnover and this strengthen its relevance to study bone biology and bone related diseases [21, 22].

The transparency of zebrafish embryos and larvae has been largely used in developmental studies to directly follow in vivo organs and tissues formation, including bone, also taking advantage of the several transgenic lines available expressing fluorescent proteins under a wide range of specific promoters [23, 24].

The availability of new optimized protocols and high-resolution imaging techniques (i.e. MicroCT or Synchrotron), allow also to follow bone growth in adulthood, thus enabling the evaluation of both short and long term pharmacological therapeutic outcomes [16].

Of relevance, zebrafish has the unique ability to regenerate various tissues and organs such as caudal fins, heart, scales, central nervous system, jaw, retina, spinal cord, pancreas, liver and kidney [25, 26], providing a tremendous tool to investigate in adults in vivo both hard and soft tissues formation. For instance, fin regeneration was exploited to dissect the mechanisms underlying adverse effects of glucocorticoids (GCs), therapeutic agents commonly used to treat immune-mediated diseases [27].

Lastly, the ease of application of the reverse genetic approaches, such as CRISPR/Cas9, enabled in zebrafish the generation of several models of heritable pathologies [16]. Even if the above mention fish-specific whole genome duplication complicated teleost genetic manipulation, the investigation of the gene pairs can help to elucidate the mechanisms that underly protein structure and function as well as signal transduction cascades, genetic regulatory networks, and evolution of tissue and organ function, representing a relevant advantage for studying human diseases [28].

This review aims to provide a comprehensive overview of the available zebrafish models of OI, to discuss the possibility to use zebrafish to evaluate the skeletal and extraskeletal features of the disease and to provide an up-to-date summary of their power to dissect OI pathophysiology to pave the way for drug discovery.

## Zebrafish and OI: Skeletal and Extraskeletal Features

Collagen I is the major protein of our body, thus not only bone but also soft tissues are compromised in the onset of OI. Therefore, a deeper investigation of the disease has to be addressed by a multidisciplinary approach and the use of animal models recapitulating OI outcome is a need to properly evaluate skeletal and extraskeletal compromised tissues/organs and to develop and evaluate the efficacy of precise pharmacological treatments. Zebrafish shares with mammals the development and organization of several organs and tissues [16], thus it represents an alternative model to investigate the disease physiopathology with a comprehensive strategy (Table 1). In this section a deep description of zebrafish organs, known to be impaired in OI, will be provided underling similarities and differences with mammals and their potentiality for providing new insights on the disease.

**Table 1:** Main similarities and differences between zebrafish and mammals skeletal tissues, skeletal muscle, heart and skin

	Embryonic origin	Cells	Gene expression	Tissue anatomy
Skeletal tissue				
*d. rerio*	Cranial neural crest origin for skull; paraxial mesoderm origin for axial skeleton. Endochondral, intramembranous, perichondral and perichordal ossification	Osteoblasts/cytes, osteoclasts (mono- and multinucleated) chondroblast/cytes	*col10a* and *sox9* expressed by both chondrocytes and osteoblasts	Skull formed by 73 bones, vertebral column by 30–32 vertebrae. Lack of hemopoietic bone marrow. Lack of primary trabeculae in endochondral forming bones. Several non-pathological bone-cartilage intermediate tissues remain throughout fish life
mammals	Cranial neural crest origin for skull; paraxial mesoderm origin for axial skeleton. Endochondral and intramembranous ossification	Osteoblasts/cytes, osteoclasts (mononucleated) chondroblast/cytes	*COL10* and *SOX9* expressed only by chondrocytes	Skull formed by 22 bones, vertebral column by 33 vertebrae. Presence of bone marrow. Trabeculae and secondary ossifications in endochondral bones. Bone-cartilage intermediate tissues are transient or pathological
Skeletal muscle				
*d. rerio*	Multi-stage myogenesis from mesoderm-derived cells forming muscle fibers. Somitogenesis completed at the first day of development	Myocytes, adaxial cells (muscle development), Myocytes, SCs	*mrf4* has no role in muscle commitment *myoD* is required to drive the cranial musculature	Higher proportion of fast-white fibers. Myofibrils and sarcomere organization. Fibers divided in myomeres. Completely innerved myotome already formed during the primary myogenesis. Muscle repair involves connective tissue. Newley regenerated myofibers rarely centrally nucleated
mammals	Multi-stage myogenesis starts with mesoderm-derived cells forming muscle fibers. Somitogenesis starts at day E8 in mice and a complete muscle is visible at E13.5	Myocytes, SCs	*MRF4* controls early myogenesis. *MYOD* is not involved in cranial musculature commitment	Fast-white and slow-red fibers equally represented. Myofibrils and sarcomere organization. Fibers grouped into fascicles. Muscle innervation during secondary myogenesis. Muscle repair involves connective tissue. Newley regenerated myofibers are centrally nucleated
Heart				
*d. rerio*	Lateral plate mesoderm origin of heart. AV endocardial cells form intermediate endocardial cushions after EMT and subsequentially give rise to mature valve	Cardiomyocytes, AV endocardial cells	Developmental gene expression activation after heart injury (i.e. *raldh2*, promoting cardiac myocytes proliferation)	Only a single atrium and a single ventriculum, connected by an AV valve. Cardiac muscle is composed of trabeculae projecting radially into the lumen of the chamber. Strong regeneration ability reliable on cardiomyocytes re-entering the cell cycle, also after multiple injuries
mammals	Lateral plate mesoderm origin of heart. AV endocardial cells form intermediate endocardial cushions after EMT and subsequentially give rise to mature valve	Cardiomyocytes, AV endocardial cells	Development genes expression patterns are not activated after heart injury	Four-chambered heart with two atria and two ventriculi. Two AV valves. Cardiac trabeculae characterizing embryo heart undergo compaction during development. Very limited cardiac regeneration, reliable on cardiac stem cell
Skin				
*d. rerio*	Dorsal ectoderm origin of epidermis, mesoderm origin of dermis and hypodermis	Specialized cell types (mucous goblet cells, club cells, sensory cells), basal cells	Dermal collagen I is produced by basal keratinocytes in the acellular dermis phase during embryogenesis. When fibroblasts invade the dermis, they take over the collagen I production and deposition. Collagen III is not express	Three-layers epidermis (surface, intermediate and basal). External epidermal layer characterized by living cells sink in an anti-bacterial rich mucus which are individually replaced when dead. Dermis and hypodermis are present. Dermal ECM is organized in orthogonal layers of collagen fibrils. Skin completely regenerates after wound
mammals	Dorsal ectoderm origin of epidermis, mesoderm origin of dermis and hypodermis	Keratinized cells; specialized cell types (mucous goblet cells, sensory cells), basal cells	Fetal skin is initially composed of collagen III, and during development a collagen III- to I shift occurs	Four-layers epidermis (basal, spinous, granular and corneum). Corneum layer characterized by periodically replaced cells. Dermis and hypodermis are present. Follicles and sebaceous glands. Dermal collagen fibrils are not oriented. Scar formation occurs after massive wound

*SCs* satellite cells, *AV* atrio-ventricular, *EMT* epithelial-mesenchymal transition, *ECM* extracellular matrix

### Skeletal Tissues

The zebrafish skeleton can be generally categorized into craniofacial, including parietal bones, jaw, and opercles and axial skeleton, comprising the vertebral column, ribs, intermuscular bones, unpaired dorsal, anal, and caudal fins [29]. Generally, skeletal cell types are conserved in zebrafish, with chondroblasts/cytes and osteoblast/cytes deriving from mesenchymal stem cells and osteoclasts deriving from the monocytes fusion [21]. Despite small morphological differences, skeletal cells functions are conserved between zebrafish and mammals and they share gene expression with few peculiarity (i.e. *sox9* and *col10*) [30]. For instance, in mammals *Sox9* is expressed in all chondroprogenitors and differentiated chondrocytes and plays essential roles during endochondral bone formation [31]. On the other hand, zebrafish sox*9a* and *sox9b* orthologues are required for both cartilage-replacement and dermal bones [32]. In contrast to mammals, where *collagen 10* is an exclusive marker for hypertrophic chondrocytes [33], *col10a1* expression in teleosts is also found in early and mature osteoblasts prior to mineralization and partially overlapping with osterix (*osx*) expression [34].

In zebrafish the mineralization starts from 4 to 5 dpf and three bone ossification types are described, namely intramembranous, endochondral and perichondral. The two first are shared between teleosts and mammals although with some peculiarities, whereas the latter is only described in teleost. The intramembranous ossification originates from the direct condensation of mesenchymal stem cells that differentiate into osteoblasts, deposit bone matrix and finally mineralize. Endochondral ossification requires a cartilage template that calcifies and it is subsequentially replaced by bone [16]. In the ceratohyal and the radials in the pectoral fin, type I endochondral ossification takes place in a process resembling the mammalian one, characterized by resting, proliferation and hypertrophic zone, followed by a zone where chondroclasts degrade the cartilaginous matrix. Finally, osteoblasts lay down bone matrix. In the hyomandibula, branchial arches, ethmoid and hypuralia type II endochondral ossification takes place, for which calcification and ossification zones are absent and the cartilage template is replaced by adipocytes [16]. Tubular concave bones are filled with adipose tissue, since zebrafish lacks bone marrow and hematopoietic stem cells seed the kidney [35, 36]. Despite the conserved role of cartilage as a template in bone formation, a wide spectrum of cartilage types has been reported in adult teleosts, with features that can also overlap other tissues (i.e. bone) [22]. For example, one of the most studied intermediate skeletal tissues in teleosts is chondroid bone, which is formed by chondrocyte-like cells surrounded by a bone-like matrix [37]. This heterogeneous tissue is usually considered as transient or pathological in mammals, while it is physiologically present in the mature zebrafish skeleton [22]. Therefore, care must be taken when analyzing cartilage in zebrafish, especially in adults. However, the cell types, the genetic pathways that regulate cartilage development and composition are remarkably conserved between teleosts and humans.

#### Craniofacial Skeleton

As in other vertebrates, like mice, most of the zebrafish craniofacial skeleton elements develop from the cranial neural crest [38, 39]. The zebrafish skull includes 73 bones, contrary to the 22 present in mammals, likely due to the progressive fusion of skeletal elements during evolution [40, 41]. Despite the differences in number, bony structures in zebrafish have clear homologs in mammals. In the viscerocranium, pharyngeal arches give rise to the lower part of the skull, composed of the lower jaw, the palate and ceratohyal and hyomandibular bones. Contrarily to mammals’ posterior arches that form laryngeal cartilage, zebrafish have ceratobranchial cartilages, which support the gill tissues [42]. In addition, the anatomical structure of the skull vault and its cranial sutures are conserved between human and zebrafish. The sutures are patent in early development, allowing skull and brain to grow, but while they close in human during early childhood, they never do in zebrafish. Nevertheless, knock out zebrafish that model a congenital form of craniosynostosis have been generated and exploited to elucidate the selective requirement of *tcf12* and *twist1b* in coronal suture formation to gain a better understanding of the developmental basis of suture loss in Saethre-Chotzen syndrome [43].

As part of the craniofacial skeleton the lower jaw development is regulated by Endothelin-1 signaling, a conserved pathway between mammals and zebrafish. Indeed, loss of *Edn1* severely impairs Meckel’s cartilage and ceratohyal bone formation in fish and in mice [44]. In human, *EDN1* mutations are responsible for the auriculo-condylar syndrome (ACS), a syndromic condition affecting craniofacial development, and particularly ears and lower jaw development [45]. In addition, zebrafish jaw is able to regenerate after damage, a process that involves hybrid cartilage-bone tissue (i.e. chondroid bone) and a transient cartilaginous callus formation [46].

Despite the differences the core pathways and bone development in zebrafish resemble the ones in mammals. Furthermore, the differences from mammals make this model a powerful tool to provide more insights into gene function [30].

#### Axial Skeleton

Zebrafish axial skeleton derives from paraxial mesoderm, as in mammals. Nevertheless, mammals vertebral centrum formation is determined by somites along the spine [47], while in teleosts the notochord plays a key role in the vertebral column segmentation [48]. Particularly, mammalian vertebra is built on a cartilaginous precursor characterized by endochondral ossification, while zebrafish vertebral centra form through direct intramembranous ossification. In contrast to mammals, they are filled with vacuolated notochord cells and become surrounded by adipose tissue [49]. Zebrafish vertebral bodies do not contain bone marrow and adult hematopoiesis takes place in the kidney. Moreover, zebrafish vertebrae have trabecular bone even if it is micrometer-thin and found surrounding the narrow centra [37]. These discrepancies need to be considered when the zebrafish is used as a model for vertebral investigation in diseases. Nevertheless, zebrafish vertebral column is largely used in the field of bone research to evaluate bone quality parameters in both physiological and pathological conditions. Indeed, zebrafish share almost the same number of vertebrae (30 to 32 in zebrafish versus 33 in human) and the same physiological curvature with human, namely kyphosis in the abdominal region where the ribs protect the viscera and lordosis in the caudal region [50]. As in human, the natural body curvature in teleosts can be emphasized by environmental, nutrition, physical injury or genetic factors and malformation on vertebral column can be easily detectable [51]. Indeed, several scoliosis models have been generated by both forward and reverse genetic approaches [52]. The spine curvature alterations or deformities have been used as a parameter to systematically screen adult zebrafish models of bone diseases generated through ENU mutagenesis [29, 53, 54]. Spinal dislocation is a well-established parameter for investigating aging since backbone deformities, together with vertebrae shape and tissue alterations, are often reported in older fish [55].

Compared to mice, for which gravitational forces apply orthogonally to the spine, zebrafish endure cranial to caudal spinal loads that are originated by swimming forward through water coupled with caudal propulsion and that are quite comparable to the gravitational load to which human are subjected. Thus, by exploiting a swimming chamber, it was possible to increase the forces acting on zebrafish spine mimicking musculoskeletal exercise [56]. Indeed, several OI zebrafish, but not murine models, are characterized by vertebral compressions and fractures, making them the best models to dissect the impact of the disease on column properties in adults [53, 57–61].

#### Appendicular Skeleton

The appendicular skeleton of zebrafish and human include pectoral (shoulder) and pelvic (hip) girdles with associated appendages: fore- and hindlimbs in human, pectoral and pelvic fins in zebrafish. Human limbs originate from endochondral bones, whereas zebrafish paired fins consist of set of intramembranous bone supported proximally by endochondral radial bones. In human, most of the pectoral and pelvic girdles are also endochondral, although portions of the clavicle and scapula are formed by intramembranous ossification. Similarly, the zebrafish pectoral girdle contains a mixture of intramembranous and endochondral bones, while the pelvic girdle is exclusively endochondral [62]. The expression pathways underling fins and limbs development are generally conserved. Hox genes are required for normal appendage patterning in tetrapods [63] and in teleosts, where *hoxa13a*^−/−^; *hoxa13b*^−/−^ zebrafish mutants fail to form distal fin rays [64]. Beyond pectoral and pelvic fins, zebrafish possess a caudal fin that has no equivalent structure in human, but it has been widely exploited in bone research thanks to its regeneration ability that recapitulate in adult embryonic bone formation. After amputation caudal fin starts to regenerate through a proliferative mass of fibroblasts and osteoblasts able to dedifferentiate to lineage-restricted progenitors and accumulate in the amputation region, forming the blastema. Then, proximal cells begin the differentiation process while the proliferative compartment is maintained in the distal region. Finally, the blastema is totally replaced by differentiated cells and in two weeks caudal fin is fully regrown [65]. The caudal fin regeneration ability helped to dissect the cellular mechanisms involved in osteoporosis induce by glucocorticoids [66]. Also, it has been exploited to investigate the effect of 4-phenilbutyrate (4-PBA), an anabolic drug already demonstrated to improved mineralization and collagen I secretion in dominant and recessive form of OI [67–69]. An increased percentage of regenerated caudal fin rays and caudal fin surrounding tissue was observed after 4PBA administration in adult *p3h1*^*upv2/upv2*^ zebrafish model, confirming the positive effect of 4PBA on cellular homeostasis and particularly on bone formation [70]. Therefore, caudal fin regeneration studies represent a valid tool for the identification of new therapeutic approaches, by exploiting adult fish in a minimally invasive way.

#### Scales

Scales, absent in human, are mineralized structures of the zebrafish exoskeleton, characterized by an external calcified layer and an inner collagen I-rich plate, both containing osteoblasts. Moreover, the expression of both Cathepsin k and Rank, two of the main markers of bone resorption, revealed the presence of osteoclasts as well [71]. Scales are small, abundant and can be easily removed from adult zebrafish without sacrifice the animal, thus representing a rapid and non-invasive tool for high-throughput drug-screening [72]. For instance, alizarin red and calcein double staining on zebrafish scales allowed in vivo visualization of mineralization defects induced by prednisolone (PN) [73]. This glucocorticoid-induced osteoporosis zebrafish model was used to assess the effect of alendronate, a well-known bisphosphonate. Recently, transcriptome analysis revealed a high level of conservation in gene expression profile between scales and mammals’ endoskeleton. Among the others, a group of genes involved in OI physiopathology has been found, such as *COL1A1*, *COL1A2*, *BMP1*, *CRTAP*, *SP7* [74]. Interestingly, de Vrieze developed a scale assay exploiting a *sp7* transgenic zebrafish in order to screen osteogenic compounds acting on WNT-signaling. This quick, sensitive, and quantifiable tool allowed the identification of 3 compounds out of 77 tested which positively stimulated osteoblast activity [75]. Thus, both scales regeneration and the *ex-vivo* scales culture represent promising tools to identify novel therapeutical target for bone diseases, including OI.

#### Teeth

The dentition in zebrafish is replaced continuously throughout life [76]. The general teeth structure, components, and cells are conserved between zebrafish and mammals. Indeed, teeth are formed by a pulp cavity containing nerves and blood vessels surrounded by dentin, which is formed by hydroxyapatite mineral crystals and collagen I [77]. Dentinogenesis and amelogenesis are also conserved, as well as cell types, namely odontoblasts and ameloblasts. Therefore, zebrafish is an attractive model to study odontogenesis, tooth replacement and regeneration both in normal and pathological conditions. Indeed, OI patients are commonly affected by dentinogenesis imperfecta, a rare debilitating disorder affecting dentin formation and causing loss of the overlying enamel with high risk of tooth loss [78]. Zebrafish share several conserved pathways with mammals involved in guarantee normal dentition, such as WNT, hedgehog and TGF-β signaling [79–81]. Indeed, the ability of zebrafish to continuously form teeth throughout their lives allowed to elucidate the role of the TGF-β family member receptor Alk8 in dental epithelial patterning and in odontoblast, ameloblast and osteoblast differentiation [82]. Nevertheless, in some cases redundancy can occur in zebrafish, as observed for FGF signaling targets where additional genes are expressed in zebrafish dental epithelium, for which comparable evidence is either scarce or lacking in mammalian dentition [83].

### Joints and Ligaments

In adult zebrafish, ligaments exhibit a well-structured arrangement of collagen fibrils that is similar to that observed in mammals [84]. Zebrafish, as mammals, have skeletal joints that can be classified in fibrous joints (i.e. sutures involving skull bones), synovial joints (i.e. in the jaw and pectoral fin) and cartilaginous joints (i.e. the jaw joint between the Meckel’s and palatoquadrate cartilages) [85]. Sutures at the level of the skull are conserved between zebrafish and mammals, while synovial-like joints, observed in zebrafish articular cartilage, in the joint cavity and in the synovium lack an articular disc [86].

In teleosts, the connections of the endplates of vertebral bodies are similar in design and function to mammalian intervertebral disc (IVD). Vacuolated notochord cells centrally located with the same function as the mammalian nucleus pulposus are surrounded by a strong composite ligament that functions like annulus fibrosus [87]. From inside to outside the ligament is composed of collagen II, elastin, and collagen I fiber bundles [88]. In adult teleosts, vacuolated notochord cells turn into fibrous, keratinized, connective tissue and extracellular vacuoles [87]. Of note, IVD degeneration (IVDD), reported in OI type XII individuals carrying *SP7* mutations, was described in aged and in the *sp7* mutant zebrafish, which are characterized by altered notochord cells and fibrosis. Moreover, disc calcification, which is correlated with premature human IVDD, was observed in Cathepsin k mutants, indicating that both low and high bone mineral density contribute to increment the IVDD risk in zebrafish [89].

Ligament laxity and hypermobility has been proposed as a non-osseous feature that plays a role in scoliosis development in OI, one of the most frequently reported secondary feature of the disease. Nevertheless, no correlation was demonstrated between the severity of spinal phenotype and the presence of ligament laxity in OI patients [90]. Thus, the role of this complication in the pathogenesis of OI can be investigated by exploiting zebrafish as a model to study joints and ligaments.

### Skeletal Muscle

Zebrafish musculature is a highly organized contractile system that sustains the body architecture, and it is composed of cephalic, trunk and appendicular muscles. Trunk and tail musculature, responsible for fish locomotion, is very developed compared to craniofacial muscles that are only associated to the mouth, eye and gill movements [91]. Similarities and differences characterize teleost and mammalian musculature system. As in mammals, red and white fibers are present in zebrafish muscles. The red fibers are rich in mitochondria and myoglobin and responsible for slow and prolonged swimming, while the white fibers allow fast contractions [92]. Since zebrafish are highly adapted for rapid and prolonged swimming, their muscles exhibit a higher proportion of fast-twitch fibers, required for quick bursts of movement [93]. In contrast, human muscles display greater diversity, with a mixture of slow- and fast-twitch fibers, enabling a wider range of movements [94]. Myofibrils and sarcomere organization as well as the molecular mechanism involved in muscle contraction, development and maintenance, are conserved between zebrafish and mammals [91]. The main human sarcomere components, such as myosin thick filaments and actin thin filaments, as well as the M- and Z-lines can be easily detected in zebrafish [95]. Indeed, slow *myosin heavy chain 1* (*smyhc1*) zebrafish mutants, carrying mutations in the gene expressed in the first group of muscle fibers formed during myogenesis, show shorter sarcomere length that leads to a reduced swimming activity and increased lethality during larval stage [96].

Unlike human, in which muscle fibers are grouped into fascicles [97], zebrafish muscles are organized in myomeres, which are myofibers segmented muscle units along the body axis [98]. The horizontal septum subdivides the myomeres into hypaxial and epaxial muscles, which are ventrally and dorsally located, respectively [99]. As in mammals, teleosts myogenesis starts with mesoderm-derived cells forming muscle fibers in a multi-stage process that starts with somite formation. Nevertheless, some differences occur in the timing and progenitors’ specification [100]. Somitogenesis is completed by the end of the first day of development in zebrafish [101], while in mice it starts at day E8 [102] and a complete muscle is visible at E13.5 [103]. In vertebrates somites develop in a cranial to caudal direction forming the dermomyotome, a heterogeneous structure composed of both multipotent as well as fate-restricted progenitors giving rise to myocytes, smooth muscle, mitotic muscle progenitors, and also to dorsal dermis and endothelium of adjacent blood vessels [104]. In zebrafish, adaxial cells, responsible for the first myogenesis, are detectable before somite development beside the notochord and myotome formation begins only after somite formation, when adaxial cells migrate to form slow fibers [105]. Adaxial cells position suggested that notochord derived signaling hedgehog factors could direct their fate, indeed the analysis of the zebrafish mutant *sonic-you* showed that mutations in the sonic hedgehog signaling pathway affect muscle development [106]. In mammals, secondary myogenesis is generally characterized by muscle growth and maturation of the myotome and muscle innervation, while in zebrafish a completely innerved myotome is already formed during the primary myogenesis [107]. The phases of muscle damage repair are conserved between zebrafish and mammals, with connective tissue that forms after muscular injury [108]. This phase is resolved by satellite cells (SCs) characterized by *pax7* expression, which proliferate and differentiate into new myofibers [109]. *pax7a* and *pax7b* are both expressed in larval and/or adult zebrafish satellite-like cells and are implicated in repair and/or regeneration [110]. Indeed, *pax7a;pax7b* double mutants are severely deficient in newly-forming myofibers compared to controls [111]. It was recently demonstrated that zebrafish skeletal muscle can regenerate not only after major injuries. Indeed, Oudhoff et al. showed that also several myomeres efficiently regenerated within one month after wounding and that mTOR signaling is required for the replacement of connective with sarcomeric tissues. This new model of zebrafish myomere restoration may provide new medical perspectives for treatment of traumatic injuries [112].

Muscle pathology as part of the pathophysiology of OI was for the first time described in the *oim/oim* mouse model, carrying mutations in *Col1a2* gene [113] and more recently described in the Amish mouse carrying a α2(I)-Gly610Cys substitution [114]. Then, clinicians and basic scientists began to consider and investigate muscle function in OI patients. It was found that more than 80% of OI patients suffer from muscle weakness that seems to correlate with OI severity [115]. The availability of several zebrafish model for OI together with the aforementioned advantages in using zebrafish to study muscles represent an interesting opportunity to deeper dissect muscle pathology in presence of skeletal diseases.

### Heart

Contrarily to mammals, zebrafish hearth is simpler and only composed by a single atrium and a single ventriculum, connected by an atrio-ventricular valve [116]. Nevertheless, the molecular mechanisms driven the heart development are highly conserved between zebrafish and mammals [117]. Hedgehog pathway (Hh) is essential during human cardiac development and zebrafish *smoothened* mutant embryos display smaller and misshaped heart due to a reduced number of cardiomyocytes, demonstrating that Hh signaling plays an important role in directing multipotent stem cells into the myocardial lineage [118].

Cardiac defects such as valve stenosis, alteration in hemodynamics, arrhythmia and heart failure represent possible extraskeletal features in OI patients, likely due to the collagen I abundance in the myocardium [119]. It was recently reported that *COL1A1* mutation in OI correlates with larger diameters of the main pulmonary artery, larger left heart chambers and lower left ventricular ejection fraction, suggesting a relationship of cardiac abnormalities and pathogenic gene mutations in patients [120]. Myocardial hypertrophy at necropsy was described in *Col1a1*^*Jrt/*+^ OI mice at 20–25 weeks of age and transthoracic doppler echocardiography showed that *Aga2*^*severe*^ *Col1a1* OI mouse hearts at 6–11 days post-birth exhibited thickened intraventricular septa and right ventricular hypertrophy [121, 122].

Zebrafish has gained attention in recent years in the field of regenerative medicine thanks to the ability of regenerate their heart after damage both in larval and adult stage. In zebrafish heart injury induces inflammation followed by replacement of necrotic tissue with fibrotic scar that preserves heart structure integrity. Contrary to mammalian fibrosis that persists leading to a consequent reduction of the heart function, the proliferative activity of teleosts cardiomyocytes allows scar replacement and thus heart regeneration [123]. Injury surviving cardiac cells change their gene expression patterns, upregulating genes involved in embryonic heart development (i.e. *vmhc* [124], *gata4* [125]) and downregulating cardiomyocyte late-differentiation genes (i.e. *cmlc1*, *alpk3a*) [126].

Cardiac valves formation is conserved between zebrafish and mammals and it is regulated by the epithelial-to-mesenchymal transition of atrio-ventricular (AV) endocardial cells that migrate into the atrio-ventricular canal and proliferate to form intermediate endocardial cushions, which subsequentially form mature valve [127]. The availability of several zebrafish models for different mutated OI-related genes together with the availability of specific transgenic lines [128, 129] and the zebrafish heart regeneration ability could help in the investigation of this cardiovascular phenotype to genotype correlation.

### Skin

As mammals, zebrafish have epidermis, dermis, and hypodermis. Contrary to mammals, whose stratified structure of epidermis is composed of four layers (basal, spinous, granular and corneum), three layers (surface, intermediate and basal) constitute the teleosts’ epidermis. In terrestrial vertebrates the corneum layer, characterized by dead and keratinized cells, is missing in zebrafish and replaced by a monolayer of living cells sink in an anti-bacterial rich mucus. The intermediate layer is composed by various specialized cell types, while the basal layer is a monolayer of cells attached to the basement membrane via hemidesmosomes. No mammalian appendages such as follicles and sebaceous glands can be found in zebrafish [130, 131].

Dermis is the vascularized subepidermal skin layer characterized by an elastin and collagenous rich stroma produced by fibroblasts, that provides structural support [130]. Layers representing the epidermis and the dermis can be recognized as early as 1 dpf and at 6 dpf. The epidermis is composed of a two-cell layers and is separated from the underlying connective tissue stroma by a basement membrane. The dermal side is composed by collagenous stroma with fibroblastic cells with a well-developed rough endoplasmic reticulum. The major collagens present in human dermis, including collagens I, V and VI, are also expressed in zebrafish skin [132].

Contrary to the not oriented collagen fibrils of mammalian dermis, zebrafish dermal ECM is organized in orthogonal layers of collagen fibrils, reminiscent to the mammalian corneal stroma whose principal role is to contribute to the transparency of the tissue. Thus, the orthogonal organization of the zebrafish dermal ECM might contribute, in the same way, to its optical transparency [133]. Several zebrafish models were generated by exploiting forward, reverse genetic or knock down approaches proving the goodness of the model for human diseases of skin [134].

Zebrafish is able to completely regenerate its skin after injury in a rapid process that resembles the human one. In mammals, wound-healing stages involve several continuous and overlapping processes starting with blood clotting, inflammation, re-epithelialization, granulation tissue formation, and finally remodeling [135], while zebrafish lacks the blood-clotting phase [136]. Similarly to mammals, zebrafish macrophages (*lyz*-expressing cells) remain in healing wounds longer than neutrophils (*mpx*-expressing cells [137]) and inflammation precedes granulation tissue formation and vascularization [138]. Thus, the fast and blood-clotting independent re-epithelization and the remodeling of the collagenous scar allow zebrafish to completely restore its skin. OI patients are characterized by a stiff and poorly elastic skin [139], while Ehlers–Danlos (EDS) patients, an OI-related syndrome, present skin hyperintensity and lesions as a result of collagen disorganized and disoriented collagen fibers deposition into ECM [140]. The overmodification and reduced secretion of collagen I in P3H1 null mice, model of OI type VIII, are responsible for thinner and slightly less dense skin respect to wild type [141]. Considering the availability of OI zebrafish models and taking advantage of the larvae transparency as well as the rapid healing after injury in adults, zebrafish could be exploited as a model to investigate the skin formation and repair mechanisms in OI and EDS-related conditions.

## OI Zebrafish Models

**Fig. 1:**
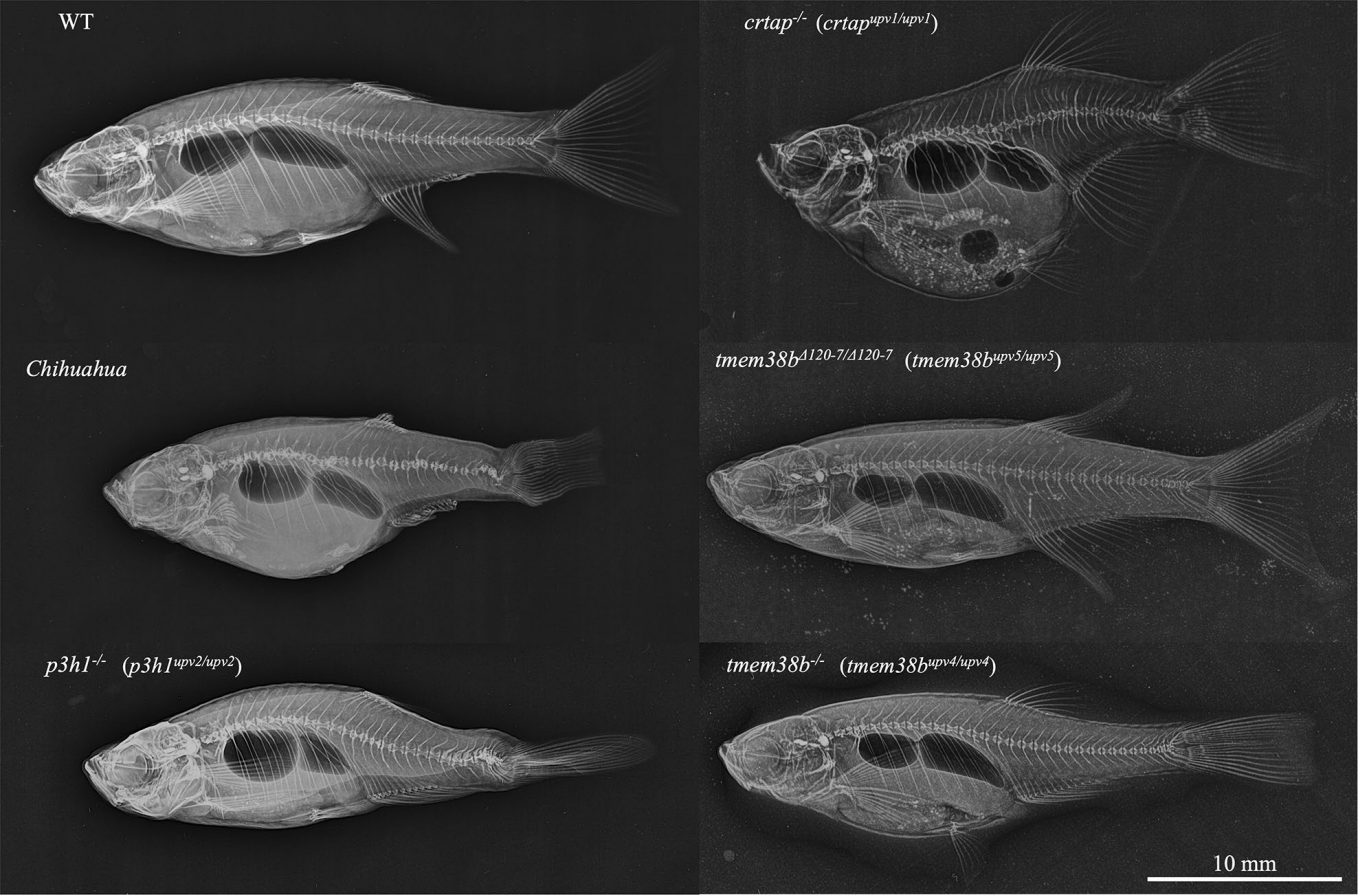
Representative X-rays of some dominant and recessive OI zebrafish models

The availability of forward genetic approaches using large chemical mutagenesis screening [29] and, more recently, the reverse genetic CRISPR/Cas genome editing technique [59, 142], applicable both to the traditional germline F2 analysis and to the more rapid-throughput F0 crispant approach [143]*,* allowed to generate several OI zebrafish mutants available to dissect the mechanisms and the pathophysiology of the disease (Fig. 1). Of interest, OI zebrafish models also reproduce the phenotypic variability described in patients [3], thus representing a unique tool for the identification of possible modifier genes that could be quickly tested taking advantage of the F0-based genetic assay.

Nevertheless, it must be considered that some OI-causative genes have more than one orthologue in teleost due to the whole genome duplication mentioned above (Table 2) [144, 145] and in these circumstances, to target all orthologues with no clear information on their transcription, translation and function properties may represent a challenge.

**Table 2:** Human OI-causative genes and their zebrafish orthologous

OI causative gene (human)	Gene location (human)	Orthologous genes (*d.rerio*)	Gene location (*d.rerio*)
*BMP1*	Chromosome 8	*bmp1a, bmp1b*	Chromosome 8, 10
*CCDC134*	Chromosome 22	*ccdc134*	Chromosome 3
*COL1A1*	Chromosome 17	*col1a1a, col1a1b*	Chromosome 3, 12
*COL1A2*	Chromosome 7	*col1a2*	Chromosome 19
*CREB3L1*	Chromosome 11	*creb3l1*	Chromosome 7
*CRTAP*	Chromosome 3	*crtap*	Chromosome 19
*FAM46A*	Chromosome 6	*tent5aa, tent5ab*	Chromosome 16, 23
*FKBP10*	Chromosome 17	*fkbp10a, fkbp10b*	Chromosome 19, 12
*IFITM5*	Chromosome 11	*ifitm5*	Chromosome 25
*KDELR2*	Chromosome 7	*kdelr2a, kdelr2b*	Chromosome 3, 12
*KIF5B*	Chromosome 10	*kif5ba, kif5bb*	Chromosome 2, 12
*MBTPS2*	Chromosome X	*mbtps2*	Chromosome 24
*MESD*	Chromosome 15	*mesd*	Chromosome 7
*P3H1*	Chromosome 1	*p3h1*	Chromosome 11
*PLOD2*	Chromosome 3	*plod2*	Chromosome 24
*PPIB*	Chromosome 15	*ppib*	Chromosome 25
*SEC24D*	Chromosome 4	*sec24d*	Chromosome 7
*SERPINF1*	Chromosome 17	*serpinf1*	Chromosome 21
*SERPINH1*	Chromosome 11	*serpinh1a, serpinh1b*	Chromosome 10, 15
*SP7*	Chromosome 12	*sp7*	Chromosome 6
*SPARC*	Chromosome 5	*sparc*	Chromosome 14
*TAPT1*	Chromosome 4	*tapt1a, tapt1b*	Chromosome 14, 1
*TMEM38B*	Chromosome 9	*tmem38b*	Chromosome 21
*WNT1*	Chromosome 12	*wnt1*	Chromosome 23

Data obtained from ZFIN [181], Ensembl 2013 [182] and Ensembl 2024 [183] databases

All the available OI zebrafish models described in this chapter have been listed on the basis of the functional gene classification [2] (Table 3).

**Table 3:** Available OI zebrafish mutants

OI zebrafish models	Mutation	Protein defect	OI type [OMIM]	Generation method	Inheritance	Major phenotypic features	Refs
Defects in collagen I synthesis and structure							
* col1a1a*^+/-^	*col1a1a* (g.1748G > T)	p.(Gly1179*)	I [166200]	ENU	AD	No skeletal abnormalities	[53]
* col1a1b*^+/-^	*col1a1b* (g.12931G > T)	p.(Cys68*)	I [166200]	ENU	AD		[53]
* col1a1a*^+/-^*; col1a1b*^+/-^	*col1a1a*; *col1a1b* (g.1748G > T, g.12931G > T)	p.(Gly1179*; Cys68*)	I [166200]	ENU	AD	Mild skeletal phenotype, low fractures frequency revealed by calli in the ribs; scoliosis, localized compression, fusions, and mild malformation of the vertebral bodies	[53]
* Chihuahua (Chi/* +*)*	*col1a1a* (g.6649G > A)	p.(Gly736Asp)	III [259420]	ENU	AD	Moderate to severe phenotype with kyphosis and scoliosis, multiple fractures	[29, 53, 57, 70]
* col1a1b*^*dmh29/*+^	*col1a1b* (g.28675G > A)	p.(Gly1123Asp)	III [259420]	ENU	AD	Kyphoscoliosis and shorter overmineralized vertebral bodies	[53]
* col1a2*^*dmh15/*+^	*col1a2* (g.15671G > A)	p.(Gly882Asp)	III [259420]	ENU	AD	Distorted and overmineralized axial and cranial skeletons, kyphosis and scoliosis	[53]
* col1a1a*^*dmh13/*+^	*col1a1a* (g.13644G > A)	p.(Gly1093Arg)	IV [166220]	ENU	AD	Bone deformities, scoliosis	[53]
* col1a1a*^*dmh14/*+^	*col1a1a* (g.14010G > A)	p.(Gly1144Glu)	IV [166220]	ENU	AD		[53]
* microwaved (med*^*−/−*^*)*	*col1a1a* (g.12013G > A)	p.(Glu888Lys)	II-IV [166210] [259420] [166220]	ENU	AR	Delayed bone ossification, scoliosis, low bone mass, low mineralized vertebrae	[53, 149]
Compromised collagen I post-translational modification							
* crtap*^*upv1/upv1*^	*crtap* (g.249_252delTTTCinsAG)	p.(Phe63Serfs*80)	VII [610682]	CRISPR-Cas9	AR	Reduced size, body disproportion, vertebral body fusions, deformities and fractures	[59]
* p3h1*^*upv2/upv2*^	*p3h1*(g.5127delCinsGGAGAA)	p.(Asp196Glufs*266)	VIII [610915]	CRISPR-Cas9	AR	Reduced size, body disproportion, vertebral body fusions, deformities and fractures	[59]
Compromised collagen processing and cross-linking							
* frilly fins (frf/*^*−/−*^*)*	*bmp1a* (g.84888G > T)	Splice site mutation leading to a deletion of 21 aa	XIII [614856]	ENU	AR	Compromised osteogenesis, bone fragility, shortened body axis and malformed craniofacial structures and fin shape	[149, 154]
* plod2*^*sa1768*^	*plod2* (g.73845 T > G/A)	p.(Tyr679*)	Bruck Syndrome/OI [609220]	ENU	AR	Decreased body length, severe skeletal deformity, fractures, overmineralized vertebrae, vertebral fusions, kyphosis and scoliosis	[53]
Altered osteoblast differentiation and function							
* sp7*^*−/−*^	*sp7* (g.40442C > A)	p.(Leu145*)	XII [613849]	ENU	AR	Lack of sutures formation, defects in bone growth and mineralization	[89, 162]
* tmem38b*^*upv4/upv4*^	*tmem38b* (g.362 _368delTGAAGGA)	p.(Lys122*)	XIV [615066]	CRISPR-Cas9	AR	Developmental delay, reduced body length	[142]
* tmem38b*^*upv5/upv*5^	*tmem38b* (g.358_381del24nt)	p.(Ala120_Thr127del)	XIV [615066]	CRISPR-Cas9	AR	Reduced vertebral length	[142]
Recently classified molecular defects							
* bulldog*	*sec24d* (C > A at the 3’ splice junction of intron 4)	Premature stop codon at aa 216	Cole-carpenter syndrome [616294]	ENU	AR	Kinked pectoral fins, shorter body length and shorter and misshapen craniofacial element	[174, 176]
* kif5blof (kif5Ba*^*ae12/ae12*^* kif5Bb*^*ae24/*+^ and *kif5Ba*^*ae12/ae12*^* kif5Bb*^*ae24/ae24*^*)*	*kif5ba* (g.284_289delTGATCG) *kif5bb* (g.277insAC)	p.(Val41_Ile42del) Premature stop codon	N/D	CRISPR-Cas9	AR (*kif5Ba*^*ae12*^ allele), AR/AD (*kif5Bb*^*ae24*^ allele)	Impaired muscle structure and chondrogenesis	[178]

Data obtained from ZFIN [181], Ensembl 2013 [182] and Ensembl 2024 [183] databases

*ENU* N-ethyl-N-nitrosourea, *CRISPR* Clustered Regularly Interspaced Short Palindromic Repeats, *AD* autosomal dominant, *AR* autosomal recessive

### OI Zebrafish Carrying Defects in Collagen I Synthesis and Structure

The first OI zebrafish models were identified, following a forward genetic approach based on N-ethyl-N-nitrosourea (ENU), either by high-throughput sequencing or by skeletal phenotypic screening of adult fish [29, 146]. The first approach allowed the identification of c*ol1a1a*^+/-^ and *col1a1b*^+/-^ mutants characterized by quantitative defects in α1(I) chain, of *col1a1a*^*dmh13/*+^, *col1a1a*^*dmh14/*+^, *col1a1a*^*chi/*+^ carrying qualitative defects in the α1 chain, as well as *col1a1b*^*dmh29/*+^ with mutation in α3 and of *col1a2*^*dmh15/*+^ characterized by qualitative defects in the α2 chain [53, 54].

*col1a1a*^+/-^ and *col1a1b*^+/-^ mutants, heterozygous for a null *col1a1a* and *col1a1b* allele, respectively, do not present any skeletal abnormalities, while the double heterozygous mutant *col1a1a*^+/-^*;col1a1b*^+/-^ displays a mild skeletal phenotype, with some cases of low spontaneous fractures, scoliosis and vertebral abnormalities, suggesting the presence of incomplete penetrance. All the three models are characterized by intra-genotype variability [53].

Qualitative collagen defects, mainly due to glycine substitutions, represent the most common cause of classical OI [1]. As generally happens for human patients, phenotypic severity is worst in zebrafish mutants carrying qualitative comparing to quantitative defects.

The *col1a1b*^*dmh29/*+^, *col1a1a*^*dmh13/*+^ and *col1a1a*^*dmh14/*+^ mutants, whose mutations result in dominant transmitted amino acid substitutions (Gly1017Asp, p.Gly1123Asp; Gly931Arg, p.Gly1093Arg and Gly982Glu, p.Gly1144Glu. respectively), are all characterized by shorter length compared to control, severe vertebral deformations, an excessive bone formation in the vertebral centrum and osteophyte generation, with a phenotype worsening in adulthood [54]. Among these mutants, *col1a1b*^*dmh29/*+^ shows the most severe phenotype, ranging from moderate to perinatal lethal, with kyphoscoliosis and shorter, thicker, and overmineralized vertebral bodies and frequent rib fractures. From a biochemical point of view *col1a1a*^*dmh13/*+^ and *col1a1a*^*dmh14/*+^ are characterized by collagen overmodification, as demonstrated by the delayed electrophoretic migration of collagen α bands, while normal collagen chains migration was detected in *col1a1b*^*dmh29/*+^ mutant.

The *col1a2*^*dmh15/*+^ mutant (Gly801Asp, p.Gly882Asp) is characterized by heavily distorted, misshapen and overmineralized axial and cranial skeleton [53], all features resembling patients’ phenotype [1]. From a biochemical point of view, collagen is not or only slightly overmodified in these zebrafish mutants [53].

The first better characterized OI zebrafish model for classical OI is *Chihuahua* (*Chi/* +), identified after ENU mutagenesis by skeletal phenotypic screening of adult fish [29]. *Chi/* + carries a heterozygous glycine substitution in the triple helical domain of α1(I) (Gly574Asp, p.Gly736Asp) [29] and recapitulates a very severe, not lethal form of OI with deformed and fragile bones, multiple rib and vertebral fractures as well as a delay in mineralization starting from larval stage [57, 147]. Due to the disorganized collagen fibers, adults’ vertebrae shape is disrupted and bone geometrical parameters are impaired [61]. Bone stiffness is reduced, while a highly mineralized tissue accumulates near the intervertebral ligament [61, 147].

From a biochemical point of view and, similarly to what observed in OI patients [68] and murine models [148], *Chi/* + collagen I is overmodified and partially retained inside the endoplasmic reticulum (ER), leading to ER-stress, as demonstrated by the presence of enlarged ER cisternae and by an increased expression of the collagen specific chaperone Hsp47 [57].

A delay in osteoblast differentiation and in bone formation as well as an increase in bone resorption are present in *Chi/* + caudal fin rays [70]. In addition, an increased number of lipid droplets characterizes adult *Chi/* + caudal fin, suggesting a skewing of mesenchymal stem cells towards adipocytic rather than osteogenic differentiation [70], as observed in the OI *Brtl* mouse [148]. Finally, *Chi/* + osteocytes lacunar volume and density are impaired, leading to less pronounced osteocyte network [61].

The *microwaved* zebrafish mutant (*med*^*−/−*^) is the only classical OI zebrafish model with a recessive mutation in *col1a1a,* which results in the amino acid substitution Glu1050Lys, p.Glu888Lys in the α1 chain. The heterozygotes (*med*^+/-^) show only a ruffled fin fold at larval stage without phenotype in the adulthood. Only the homozygotes (*med*^*−/−*^) exhibit delayed ossification at 11 dpf and a peculiar undulated larval fin at the tip of the tail. Impaired bone mineral density of both distal fin rays and vertebrae is also observed in adults [149].

### OI Zebrafish with Compromised Collagen Post-Translational Modification

During collagen I folding, procollagen chains undergo post-translational modifications, catalyzed by specific hydroxylases and glycosylases inside the endoplasmic reticulum (ER). In human, the hydroxylation in C3 of α1(I)-Pro986 is specifically catalyzed by the P3H1 complex, which exerts both an enzymatic and a chaperone activity on proα chains and acts as an inhibitor of fibril formation for the folded triple helix. The complex is constituted by cartilage associated protein (CRTAP) encoded by *CRTAP*, prolyl 3-hydroxyase 1 (P3H1) encoded by *P3H1* and cyclophilin B (CyPB) encoded by *PPIB,* present in a 1:1:1 ratio [150]. Mutations in *CRTAP*, *P3H1* and *PPIB* genes are responsible in human for OI type VII, VIII and IX, respectively [1].

The CRISPR-Cas9 gene editing was used to knock-out in zebrafish genome either *crtap* or *p3h1*. The two models are characterized by a general growth delay, as proved by a reduced fish length, and altered swim bladder inflation. Also, mineralization is delayed in both mutants starting from 1 week post fertilization (wpf) in *crtap*^*upv1/upv1*^ and from 2 wpf in *p3h1*^*upv2/upv2*^. During juvenile stage body and skull disproportions are detectable in both mutants, but the phenotype gets worse during adulthood, when severe vertebral body fusions, deformities and fractures are observed [59]. Similarly, a severe skeletal phenotype characterized by vertebral and ribs fractures was also observed in OI type VII and VIII patients, which generally have higher lethality than zebrafish models [151]. Nevertheless, accordingly with the high mortality rate of OI type VII children with null mutation in *CRTAP* [152], few *crtap*^*upv1/upv1*^ reach adulthood. Their severe phenotype is characterized by chunky head and several malformations in vertebral endplates. Due to limited availability of *crtap*^*upv1/upv1*^ adults, bone geometrical parameters were analyzed only in *p3h1*^*upv2/upv2*^, which displays reduced vertebral bone volume and thickness [59]. Moreover, a recent study on *p3h1*^*upv2/upv2*^ regeneration ability of the caudal fin reveals a delayed ray regeneration rate as well as a reduced number of rays and an increased number of segments and bifurcations. Since it was reported that the two mineralizing fronts of the forming ray are able to fuse/stitch, through an osteoblast-dependent process, and then to undergo an anti-stitching process, mediated by osteoclasts, that defines the positioning of the branchpoint [153], the increased osteoclast activity observed in *p3h1*^*upv2/upv2*^ at the level of the regenerated caudal fin suggests the presence of an altered bone turnover [70].

Both *crtap*^*upv1/upv1*^ and *p3h1*^*upv2/upv2*^ bone matrix is distinguished by irregularly arranged collagen fibers, stemming from the assemble of impaired collagen I, although collagen fibrils are thicker in *crtap*^*upv1/upv1*^ and thinner in *p3h1*^*upv2/upv2*^ compared to WT [59]. The lack of 3-hydroxylation in zebrafish collagen I made the *crtap*^*upv1/upv1*^ and *p3h1*^*upv2/upv2*^ zebrafish models unique tools to prove the relevance of the 3- hydroxylation complex as collagen specific chaperone.

### OI Zebrafish with Compromised Collagen Processing and Cross-Linking

The *frilly fins* (*frf*) zebrafish mutant, generated by ENU mutagenesis and characterized by a peculiar undulated fin, carries mutation in *bmp1a* [154]*.* BMP1 (bone morphogenetic protein 1) is a metalloprotease involved in the maturation of procollagen through proteolytic removal of the C-terminal propeptide, essential to guarantee collagen self-assembly into fibrils [149, 155]. In zebrafish two *bmp1* orthologues exist, *bmp1a* and *bmp1b*. Zebrafish *frf*^*tm317/tm317*^ homozygous mutant display a ruffled fin, reduced body length and misshaped craniofacial structure [149, 154]. In their adulthood, body and fins length are reduced and a kink at the head-trunk boundary emerges [154].

Strong delay in vertebrae ossification and during fin growth is reported. When the spine eventually ossifies several vertebral deformations and fusions can be detected. Adult fin rays are wavy, fused between each other and characterized by calli and reduced bifurcation number. *frf *^*tm317/tm317*^ display an increased vertebral bone density [60, 149], as also reported in patients affected by OI type XIII [156]. *frilly fin* fish are characterized by normally differentiated osteoblasts, but with a cuboidal shape, limiting the contact with the bone surface. Finally, mutant’s collagen fibers’ structure is altered, probably due to the collagen I C-terminal propeptide retention [149].

The *plod2*^*sa1768*^ mutant, generated by ENU mutagenesis, is a zebrafish model of Bruck syndrome (BS). The c.2037 T > A homozygous mutation in the *plod2* gene leads to the formation of a premature stop codon and to a truncated lysyl hydroxylase 2 (Lh2) protein (p.(Tyr679*)) [58]. Lysyl hydroxylase 2 (LH2) is the enzyme responsible for the hydroxylation of collagen telopeptide lysine residues.This hydroxylation directs cross-linking of collagen fibrils in the extracellular matrix, which provides stability and tensile integrity to the collagen fibrils [157]. Similarly to BS patients, zebrafish *plod2*^*sa1768*^ mutants are characterized by reduced telopeptide lysine hydroxylation that leads to abnormal collagen cross-linking, resulting in severe musculoskeletal malformations with evidence of bone fragility [58].

Loss of Lh2 function in zebrafish causes an early-onset and progressive phenotype similar to the one observed in human BS. Like BS patients, *plod2* mutant fish have a short body axis, kyphoscoliosis and compression of the vertebral column, and bowing and kinking of the ribs. Contrary to human patients, microCT analysis revealed impaired bone mass associated with an increased vertebral centra volume and increased vertebral mineral density, suggesting a hyper-mineralization condition [58, 158]. Nevertheless, *plod2*^*sa1768*^ zebrafish may be valuable to investigate some aspects of the two OI-related conditions.

### OI Zebrafish with Altered Osteoblast Differentiation and Function

Osterix (OSX) or SP7, a zinc finger-specific transcription factor predominantly expressed in osteoblasts is essential for bone formation and homeostasis, promoting osteoblasts differentiation and maturation [159]. The *sp7* zebrafish model has been generated using ENU mutagenesis within the zebrafish mutation project [160]. Mutations in *SP7* lead to OI type XII, a recessive form of the disease characterized by several cranial deformities, mild scoliosis, hyperextensibility of the interphalangeal joints and osteoporosis [159]. Contrary to homozygous *Osx* mutant mice that die in perinatal period for respiratory stress [161], *sp7*^*−/−*^ zebrafish are vital and viable [89].

A delay in mineralization starting from 2 wpf, with bones that progressively become misshaped characterizes *sp7*^*−/−*^ larvae. During juvenile stage, *sp7* mutants present scoliosis, bone growth defects, ribs fractures and specific craniofacial malformations, resembling human patients’ outcome. In addition, 3 months old *sp7*^*−/−*^ fish are characterized by a reduced BMD compared to WT siblings [89]. Transmission electron microscopy reveals that *sp7*^*−/−*^ osteoblast nuclei are rounder than WT ones, and mutant extracellular matrix vesicles are poor in hydroxyapatite. Increased expression of some early osteoblastogenic markers, such as *ihhb*, *runx2a*, *runx2b*, and impaired expression of late osteogenesis markers, such as *bglap* and *alpl*, are described in *sp7*^*−/−*^ [162].

OI type XIV is a rare form of osteogenesis imperfecta caused by defects in the trimeric intracellular potassium channel B (TRIC-B), an integral membrane protein encoded by *tmem38b* [1], necessary for the regulation of Calcium flux [163] across the endoplasmic reticulum membrane. TRIC-B null osteoblasts are characterized by inhibited Ca^2^ calmodulin Kinase II (CaMKII) mediated signaling pathway that dysregulates SMAD signaling, important for proper skeleton growth [164]. In addition, lack of TRIC-B in osteoblasts has been recently proposed to be associated to a significant increase in superoxide production in mitochondria, further supporting mitochondrial dysfunction as well to an altered cell–cell adhesion processes [165].

Two *tmem38b* zebrafish models have been generated by CRISPR-Cas9 gene editing. The *tmem38b*^*upv4/upv4*^ zebrafish model is characterized by an out of frame deletion responsible for a premature stop codon at amino acid 122, while the *tmem38b*^upv5/upv5^ results from an *in frame* deletion of 24 nucleotides that leads to the removal of the highly conserved KEV domain [142], required for the correct formation of the pore channel [166, 167]. OI type XIV patients are characterized by a wide spectrum of severity ranging from mild forms with few spontaneous fractures [168] to more severe cases affected by cardiac and pulmonary implications [169]. Interestingly, this variability is reflected in the available models of the disease. Indeed, while the TRIC-B knock out mice are lethal soon after birth [170] and the osteoblasts specific conditional knock out mice present a very severe phenotype [164], *tmem38b* zebrafish mutants are characterized by milder skeletal features [142]. Particularly, only a reduced notochord mineralization in larvae and misshaped vertebral bodies in juveniles are detectable in *tmem38b*^*upv4/upv4*^. In both zebrafish mutants the under-modification of collagen I is observed, thus reproducing the typical feature observed in OI type XIV patients [171] and conditional knock-out mice [164]. Moreover, an increased expression of the fibrillary collagen chaperone Hsp47b is detected, suggesting the presence of cell stress, likely due to an impaired collagen secretion. Indeed, compromised collagen extracellular deposition is demonstrated by the reduced length of actinotrichia, collagen-made structures at the tip of fin rays. Finally, impaired osteoblasts activity is proved by the limited mineral deposition during caudal fin regeneration [142]. Therefore, the *tmem38b* zebrafish mutants could represent valid models to investigate the role of Tric-b at cellular level.

TRIC channels together with TRIC-B also include TRIC-A encoded by *TMEM38A.* Contrary to TRIC-B that is ubiquitously distributed in the body, and coupled with inositol trisphosphate receptor (IP3R), TRIC-A is mostly abundantly present in excitable cells and coupled with ryanodine receptors (RyRs). Indeed, TRIC-A mutations have been identified in patients with stress-induced arrhythmia [172]. TRIC-A knock out mice have a normal lifespan, show abnormal sarcoplasmic reticulum (SR) Ca^2+^ handling in both skeletal and cardiac muscle cells. Moreover, TRIC-A ablation causes hypertension in mice due to altered Ca^2+^ signaling in smooth muscle [173]. No skeletal diseases associated to TRIC-A deficiency have been described so far in human. Although *tmem38a* expression has been demonstrated [142], no zebrafish model exists. In addition, the expression analysis of *tmem38a* in different *tmem38b* mutants’ tissues did not show any compensatory effect [142].

### OI Zebrafish with Defects in the More Recently Identified OI Genes

Four independent ENU-induced homozygous mutations, named *bul*^*m757*^, *bul*^*m606*^, *bul*^*m494*^ and *bul*^*m421*^, were identified in *sec24D* gene, encoding a specific component of the COPII vesicles, involved in large protein export from the ER [174]. The zebrafish *bulldog* has been characterized as model for Cole-Carpenter like dysplasia, caused by a mutation in *SEC24D* gene and presenting some of the osteogenesis imperfecta features [175]. Embryos are shorter than WT and display kinked pectoral fins and shorter and misshapen cranial elements [174, 176]. Early skeletogenesis is not affected by the mutation, while the late cartilage development is altered, resulting in a pronounced dysmorphology. Collagen II and ER-quality control pathway components are overexpressed in *bulldog* respect to WT and collagen II accumulates in vesicles within mutants’ chondrocytes. These results suggest an impairment in collagen II synthesis and processing and an induced ER-stress response in chondrocytes during craniofacial development [174]. Accordingly, in a human Cole-Carpenter patient fibroblast line the procollagen export from ER is altered and ER cisternae are enlarged [175]. Also, *bulldog* chondrocytes morphology is altered, highlighting features that are typically shown in chondroprogenitors at earlier developmental stages [174].

Recently, mutations in *KIF5B* gene have been identified in patients with a clinical diagnosis of OI. *KIF5B* gene encodes the heavy chain of Kinesin-1, a member of the Kinesin superfamily of motor proteins (KIFs), crucial for intracellular transport. *KIF5B* is expressed ubiquitously and facilitates the movement of organelles, vesicles, and proteins along microtubules within cells, playing key roles in neuronal axonal transport, cell division, endocytosis/exocytosis [177].

The *kif5blof* zebrafish is a compound mutant derived from the mating of *kif5Ba* and *kif5Bb* mutants, which have been generated by CRISPR/Cas9 gene editing. *kif5Ba* mutants are characterized by a mild, incompletely penetrant phenotype, while *kif5Bb* mutants are viable fertile adults. To analyze the effect of the *kif5B* genes loss of function, *kif5blof* compound mutants has been generated [178]. Similarly to the mouse model [179], *kif5blof* larvae display defects in trunk and tail muscle structure. Abnormal chondrogenesis in the compound mutant results in prominent jaw defects [178]. Cartilage alterations is also found in the *Col2cre;Kif5b*^*fl/−*^ conditional knock-out mouse, in which the growth delay is correlated to the defects at the long bones growth plate zones due to the specific absence of *Kif5b* in the cartilage [180]. At a cellular level, mutant chondrocytes are smaller than WT and present a roundly shape and numerous lysosomes, which cumulate in the chondrocyte cytoplasm. These data, together with the deregulation of the autophagic markers, suggest a lysosomal dysfunction that led to chondrocytes apoptosis [178]. The number of TRAP + cells is significantly increased in the *kif5blof* zebrafish mutant [178], as also found in the tibial growth plate of the *Col2cre;Kif5b*^*fl/−*^ mouse [180].

The lack of a normal cartilaginous scaffold in *kif5blof* mutants impairs perichondral ossification, which remains altered during fish development [178], thus recapitulating the hypomineralization found in patients with mutations in *KIF5B* [177].

## High- and Low-Throughput Techniques to Investigate Zebrafish Tissues in Healthy and Pathological Conditions

**Fig. 2:**
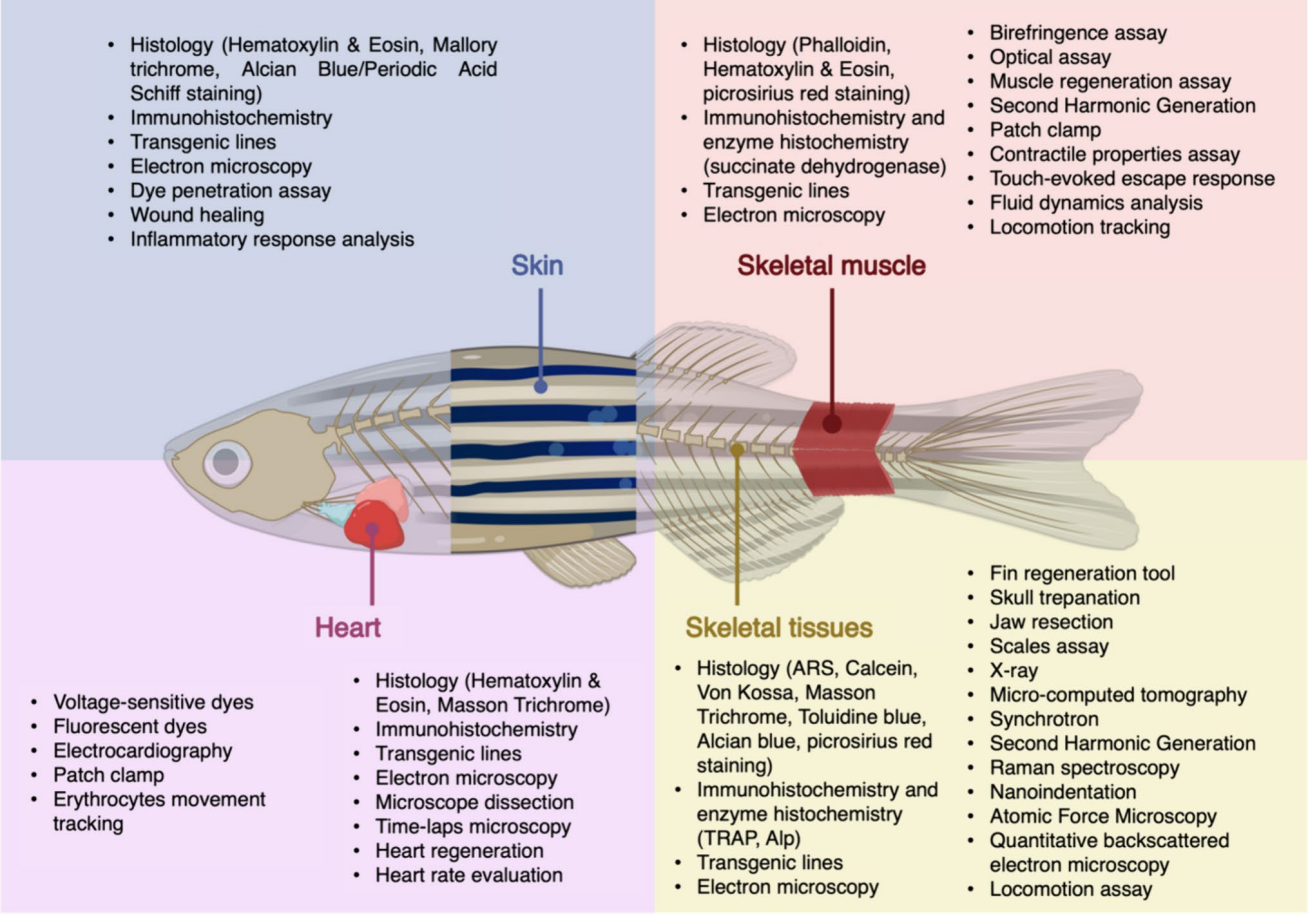
Available techniques to analyze zebrafish tissues. Created with BioRender.com

In the last decades, zebrafish became a suitable animal model in the field of biomedical research prompting the researchers to fine tune techniques to characterized skeletal and extraskeletal tissues (Fig. 2). In this review high- and low-throughput techniques are classified according to the evaluation of the quantity and timing of sample processing and according to their potential adaptation to higher throughput platforms. A brief description is provided below and summarized in Table 4. Few examples are also provided.

**Table 4:** Main available techniques to analyze OI-related zebrafish skeletal and extraskeletal tissues

Tissue	Parameter	Techniques	Life stage	High throughput?
Skeletal tissues	Bone morphology and composition	Toluidine blue	L + A	No
		Masson–Goldner trichrome	A	No
		Picrosirius red staining	L + A	No
		Electron microscopy	L + A	No
		Enzyme histochemistry (TRAP, Alp)	L + A	No
		Immunohistochemistry	L + A	No
		Transgenic lines	L + A	Yes
	Bone formation	ARS/calcein double staining	A	No
		ARS/alcian blue double staining	L + A	No
		Caudal fin regeneration	A	Yes
		Scales regeneration	A	Yes
		Jaw regeneration	L + A	No
		Teeth regeneration	L	No
	Mineralization	Alizarin red (ARS)	L + A	Yes
		Calcein staining	L + A	Yes
		Von Kossa staining	A	No
		X-ray	A	No
		MicroCT	A	No
	Bone geometrical parameters	MicroCT	A	No
		Synchrotron	A	No
	Mechanical properties	Nanoindentation	A	No
		Atomic force microscopy (AFM)	L + A	No
	Bone matrix composition	Raman spectroscopy	L	No
		Fourier transform infrared spectroscopy (FTIR)	A	No
		Quantitative backscattered electron microscopy (qBEI)	A	No
	Swimming behavior analysis	Locomotion assay	A	Yes
Skeletal Muscle	Skeletal muscle morphology and composition	Histology and immunohistochemistry	L	No
		Birefringence assay	L	No
		Electron microscopy	L + A	No
		Bright field microscopy	L	Yes
	Skeletal muscle formation	Transgenic lines	L	Yes
		Muscle regeneration	L + A	No
	Skeletal muscle functionality	Patch Clamp	L	No
		Contractile properties assay	L	No
		Calcium flux analysis	L	No
	Skeletal muscle performance	Locomotion assay	L	Yes
		Touch-evoked escape response	L	Yes
		Fluid Dynamics analysis	L	No
Heart	Heart morphology and composition	Microscope dissection	A	No
		Myocardial transgene zebrafish lines	L + A	Yes
		Electron microscopy	A	No
	Heart formation	DsRed or GFP expressing cardiomyocytes	L	No
		Time-lapse confocal microscopy	L	No
		Heart regeneration	L + A	No
	Heart contractile function	Heart rate evaluation	L + A	Yes
		High-speed video microscopy	L	No
		Erythrocytes movement tracking	L	No
	Cardiac conduction function	Voltage-sensitive or fluorescent dyes/transgene	L	No
		Electrocardiography	A	No
		Patch clamp	L	No
Skin	Skin morphology and composition	Histology and immunohistochemistry	L	No
		Transgenic lines	L	Yes
		Electron microscopy	L	No
	Skin barrier function	Dye penetration assays	A	No
		Wound healing	A	No

*L* larva, *A* adult

### High-Throughput Techniques

**Fig. 3:**
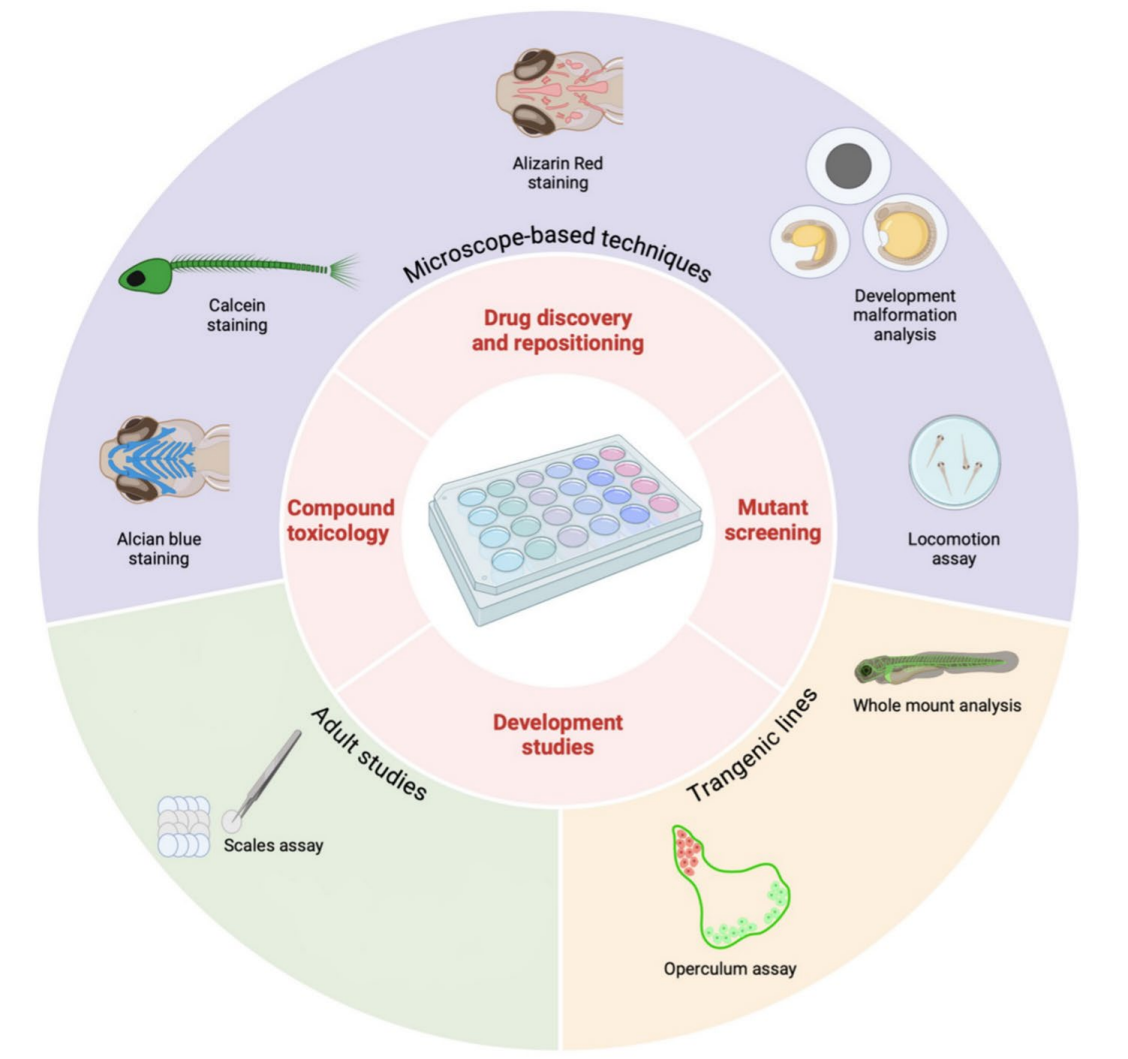
High-throughput techniques to analyze zebrafish skeletal tissues. Several techniques can be performed to analyze large zebrafish samples for developmental, for mutant screening, for studies after pharmacological treatment and to assess compound toxicity. Direct inspection under light or fluorescence stereomicroscope can be facilitated by skeletal tissue stainings such as alcian blue, calcein and alizarin red staining. Transgenic lines can be exploited for whole mount analysis or specific well-established assays, such as the operculum analysis. High-throughput analysis on adult fish involve scales studies. Created with BioRender.com

The large number of embryos from a single mating, the larvae transparency and the possibility to screen several samples at the same time drastically reducing time and costs, make zebrafish embryo and larvae unique models for the application of high-throughput analyses (Fig. 3).

#### Whole Mount Stainings

Whole mount vital stainings are often used to visualize tissues during development or to investigate the effect of pharmacological treatments, by exploiting larval body clarity. Bone development can be easily followed in vivo by calcein and alizarin red staining [184, 185]. For instance, calcein staining was used to analyze zebrafish vertebrae mineralization to screen bone-active compounds at increasing doses [185]. Alizarin red (ARS) staining allowed to investigate the tigecycline and minocycline efficacy in zebrafish larvae with prednisolone-induced osteoporosis [186]. Both ARS and calcein stainings have been used to determine the impaired mineralization of the zebrafish model with high iron stress-induced osteoporosis and to test alendronate goodness to rescue the phenotype [187]. Moreover, in the OI field the delayed mineralization in *Chihuahua*, *p3h1*^*upv2/upv2*^ and *crtap*^*upv1/upv1*^ mutant larvae and the effect of the 4PBA chemical chaperone on *Chi/* + cranial bone mineralization have been assessed through ARS [57, 59].

#### Transgenic Lines

Nowadays, several transgenic lines carrying different fluorophores under specific promoters have been generated to overcome the technical limit posed by low zebrafish antibody availability. Thanks to these models several disease related pathways have been dissected or new cell lines specificity identified. For instance, the *osterix*:mCherry and *entpd5*:YFP double transgenic zebrafish were generated to demonstrate the specific expression of the ectonucleoside triphosphate/diphosphohydrolase 5 (*entpd5*) in pre-osteoblasts [188]. *sp7*:mCherry zebrafish line has also been crossed with *oc*:EGFP fish in order to analyze osterix and osteocalcin expression in zebrafish opercle after calcitriol and cobalt chloride exposure [189]. The *ins:nfsb-mCherry* transgenic fish displaying diabetes-induced osteoporosis have been used to test the effect of cinacalcet and paricalcitol treatment, revealing an increased mineralization area in the operculum as well as an improvement in the caudal fin regeneration ability compared to controls [190]. A more exhaustive list of transgenic zebrafish tracing bone cells and/or pathway can be found in [16].

Many zebrafish reporter lines targeting the skeletal muscle are also available, as reported in [91]. For instance, the *mylz2*:GFP and the *smyhc1*:GFP lines allow the identification of fast [191] or slow [192] skeletal muscle fibers, respectively.

Quiescent and proliferating cardiomyocytes can be easily distinguished in vivo under fluorescent microscope thanks to the *cmlc2:*FUCCI zebrafish transgenic line that expresses red or green fluorescent ubiquitination-based indicator (FUCCI) according to cell cycle phase [193].

Crestin is physiologically expressed until 72 hpf, and then it remains silenced during zebrafish subsequential stages of life. Therefore, *crestin:*EGFP transgenic fish can be exploited to visualize neural crest progenitors during development as well as melanoma cells in adults [194].

#### Drug-Screening Platform

The ability of zebrafish larvae to absorb molecules through their mouth and gills allows to screen several compounds directly dissolved in fish water and treat a huge number of samples. Then, malformation development (i.e. embryo coagulation, lack of somite formation, non-detachment of the tail and lack of heartbeat) can be rapidly detected by bright-field microscopy [195]. Also, larval locomotion assays have been developed as high-throughput tools to study behavioral alteration on zebrafish larvae. Indeed, zebrafish embryos movement can be monitored using a recording chamber equipped with an infrared digital camera, allowing the analysis of multi-well plates with a larva per well. The quantification of swimming behavior parameters, such as the total swim distance and the number of movements made by each fish, is automatically performed by dedicated software, such as Stream Pix5 [196].

Semi-automated massive chemical screenings have been generated to reduce the risk of introducing bias from the operator. Functional and morphological parameters such as heart rate, arrythmia and ventricle diameter are registered taking advantage of a semi-automatic technology developed by Dyballa and colleagues to evaluate the effect of cardiotoxic compounds [197].

Recently, new and more rapid approaches for drug screening have been also optimized using juveniles and adult zebrafish. Indeed, at 11 mm the juveniles have developed most of the adult morphology [198] and physiology and can still be kept in 24-well plates, requiring small volumes which allow for reasonable screening throughput and low cost. This approach was used to elucidate the role of NF-kB during osteoblast dedifferentiation and to identify an unexpected role for NF-kB signaling in maintenance of the differentiated state of osteoblasts [199].

More challenging is the application of chemical screens to adult, up to now limited by the additional demands of cost, space, and labor associated with the screens. Nevertheless, thanks to the 3D printed system ScreenCube 96 fish can be treated in ∼3 min with a tenfold reduction in drug quantity compared to that used in previous chemical screens in adult zebrafish [200].

#### Behavioral Assay

Behavioral changes in animals could be influenced by age, strain [201], drug exposure [202] or could reflect functional alterations in organs and tissues, and they are largely studied in zebrafish larvae as a tool for drug screening [203] or tissue functionality [196]. In recent years, several systems to study swimming performance on adult zebrafish have been developed.

These systems were exploited to study the effect of exercise on zebrafish bone formation and mineralization. Fish subjected to exercise through a swimming chamber display increased bone geometrical parameters values and higher osteoblast numbers and dimension. More recently, the swimming behavior of the dominant OI zebrafish model *Chihuahua* has been tracked, revealing an altered head meandering and an impaired traveled distance [70].

### Low-Throughput Techniques

Zebrafish research is not only restricted to embryo and larval stages studies, but also adult represent a valid tool in the field of disease mechanisms evaluation. Indeed, thanks to the zebrafish easy genetic manipulation several models have been generated by reverse genetic approaches [16]. In addition, since zebrafish have a longer life span compared to rodents, they became also suitable in the field of progressive worsening diseases and in aging. Moreover, the small dimensions of adult zebrafish respect to mammals allow to increase number of samples and thus the statistical power of data, providing first evidence of the positive or negative effects of specific treatment. Therefore, in the last years several techniques have been down-scaling to allow adult zebrafish models characterization [204–206].

#### Regeneration Studies

The ability of adult zebrafish caudal fin to regenerate in only 14 days allows not only to follow bone formation [207] and repair after fracture but also to evaluate pharmacological therapies. Indeed, caudal fin regeneration ability is impaired in osteoporosis-induced zebrafish models [190, 207] as well as in dominant and recessive OI zebrafish mutants [70], and thus they have been exploited to test promising pro-osteogenic compounds [70, 190]. Moreover, fracture repair mechanisms have been characterized in zebrafish caudal fin. In the *frf* zebrafish mutant, characterized by spontaneous fractures in the lepidotrichia, ARS/calcein double staining revealed an impaired bone callus architecture [149]. Alendronate administration in *frf* fish reduced spontaneous fracture rate, while it alters bone calli resorption in WT fish [208].

#### Histological Staining

As for mammals, adult zebrafish histology is one of the most common used techniques, since it provides several information concerning cells morphology, distribution and activity and tissues microarchitecture. Also, in zebrafish the sectioning of mineralized tissues requires decalcification protocols or methacrylate-based polymer embedding. However, some dyes can be used in whole mount approach also in adults, such as ARS, which empowers bone visualization through rhodamine-filtered fluorescence microscopy [209]. The most employed skeletal tissues staining are the Masson’s trichrome and toluidine blue generally performed to visualize bone morphology [56, 89], alizarin red and calcein to evaluate the mineral apposition rate [210] and von Kossa to analyze mineralized tissues [147] and sirius red staining to visualize collagen fibers maturation under polarized light [211]. Von Kossa staining showed highly mineralized endplates with no osteoid in *Chi/* + compared to WT, and the low phosphorous diet restored the osteoid in mutants, reaching WT levels [147]. Picrosirius staining revealed a higher amount of collagen in the caudal fin regrowth matrix of *Chi/* + fish after 4PBA treatment compared to control fish [57]. To investigate osteoclast activity and bone ECM mineralization functionality tartrate-resistant acid phosphatase (TRAP) and alkaline phosphatase (ALP) activity assays can be performed. These stainings allowed to characterize the effect of alendronate on scales of a glucocorticoid-induced osteoporosis (GIOP) zebrafish model [73, 212]. Importantly, transgenic samples maintain specific fluorescent signals during embedding and sectioning, thus allowing detailed high-resolution imaging also in adult zebrafish, that are not any longer transparent [213].

Zebrafish muscular tissue can be visualized by classical staining such as hematoxylin eosin (H&E), while muscle alteration myofiber structure can be detected under polarized light taking advantage of muscle anisotropy coupled with zebrafish larvae transparency. For instance, *col6a1* morphants, model of collagen VI-related myopathy, display an abnormal birefringence that is not improved by cyclosporin A treatment [214].

Acid fuchsin orange G staining, which labels cardiomyocytes in orange, fibrin in red and collagen in blue, was useful to demonstrate that the fibrin-rich area, corresponding to the fibrotic tissue that forms after zebrafish heart injury, is strongly reduced by 14 days post injury (dpi) while a collagenous region is still present at 60 dpi [215].

H&E staining was also used in zebrafish skin regeneration studies to investigate the effect of Fgf disruption and hydroxyurea treatment on cell proliferation [216].

#### Electron Microscopy

Scanning (SEM) or transmission (TEM) electron microscopy provide high-resolution surface imaging or ultrastructural details of zebrafish, respectively. SEM is particularly suitable to describe structures morphology, and it was indeed performed to detect dose-dependent simvastatin-induced alterations in zebrafish embryos tail. SEM can also be used to study zebrafish cutis during development or infection since cells (i.e. keratinocytes) and other cutis element (i.e. scales) can be imaged [217]. Zebrafish cells are typically smaller than mammalian cells, especially during early developmental stages. Thus, TEM can provide detailed information that are not easily detectable through classical histological analysis. TEM allows visualization of fine structural features such as collagen fibrils [57, 147], myofibrils or sarcomere organization [218, 219] and cellular organelles such as endoplasmic reticulum or nuclei [57, 147]. Indeed, TEM analysis on aged scoliotic zebrafish revealed severe myofiber disorganization and degeneration, thus contributing to underline the close biomechanical relationship between bone and muscle [220]. Also, enlarged endoplasmic reticulum cisternae was found in the osteoblasts of *Chihuahua* [57], *p3h1*^*upv2/upv2*^ and *crtap*^*upv1/upv1*^ [59] zebrafish models through TEM, confirming ER-stress as a possible pharmacological target for both dominant and recessive OI. Indeed, the positive effect of 4PBA, a molecular chaperone known to reduce ER-stress, has been confirmed in *Chi/* + fish by TEM images [57].

#### X-ray Based Imaging Techniques

X-ray imaging is a cheap and quick technique to visualize the zebrafish skeleton. It can be repeated on live organisms and it can be used as a preliminary diagnostic tool for skeletal defects characterization. An intermediate throughput power can be attributed to X-ray considering its role in screening approaches to identify skeletal mutants in forward genetic studies [29]. Although x-ray imaging can be employed to assess skeletal deformities in adult zebrafish, its use for juvenile zebrafish, where the skeleton is too small to be captured on film or digitally, is not feasible. In addition, x-ray images of zebrafish are not suitable for quantification of tissue or bone mineral parameters compared to the high-resolution microCT. Micro computed tomography (μCT) is an x-ray-based technology that enables *ex-vivo* examination of the complete zebrafish skeleton through the reconstruction of a 3D image. Medium and high-resolution scans on zebrafish samples can be acquired using a 21 μm [60] or a 10.5 to 5 μm voxel size [57, 59, 60], respectively. In particular, the use of low resolution microCT in semi-throughput platform allowed many fish to be examined in multiplex and the development of microCT-based phenomics for a deep skeletal zebrafish phenotyping [60]. Thanks to μCT, compromised bone volume, vertebral thickness and bone mineral density have identified in the dominant OI zebrafish model *Chihuahua*. μCT analysis can also be performed on soft tissues by using contrast-enhancing staining methods [221]. Indeed, scanning of phosphotungstic acid (PTA)-stained fish allows soft tissue visualization and subsequential 3D rendering of eyes, heart and brain.

Higher-resolution images can also be obtained by synchrotron X-ray tomographic microscopy, which can also distinguish mineralized and non-mineralized bone. Indeed, a 0.65 μm voxel size has been assessed to demonstrated that a low- and high-phosphorous diet, respectively increase the non-mineralized and mineralized matrix in zebrafish vertebrae [204].

The application of high-resolution synchrotron μCT images allowed to demonstrate the presence of fewer osteocytes in the lacunae of the *sp7* zebrafish mutant respect to WT.

#### Imaging and Spectroscopy for the Analysis of Matrix Composition

Second Harmonic Generation (SHG) imaging is often used to observe the striated skeletal muscle of living zebrafish as well as collagen fibers orientation and thickness without employing exogenous labels and eliminating concern about the distortion of structures caused by sample preparation in conventional histological examination [222]. SHG was also exploited to characterize collagen fibers orientation, distribution and thickness in both aged and *sp7* mutant zebrafish [89].

Quantitative backscattered electron imaging (qBEI) allows to analyze the inorganic composition of bone matrix. Indeed, backscattered electrons interact with the sample and produces a signal intensity that depends on the local mean atomic number of the sample itself. qBEI helped to detect the hyper mineralized bone tissue area of the dominant OI zebrafish *Chihuahua* [61].

Other techniques based on vibrational spectroscopy methods, such as Raman spectroscopy (RS) and Fourier-Transform Infrared spectroscopy (FTIR), provide information on both inorganic and organic bone components, discerning tissue components based on their energy-specific vibrations [223, 224]. Confocal Raman imaging identifies specific molecular components of bone tissue, such as collagen, hydroxyapatite, and other mineral phases, offering insights into bone quality and maturation [225]. Using this approach, Høgset and collogues determined in vivo the biomolecular composition of a whole zebrafish embryo, discerning collagens, lipids, DNA and cytochrome-rich regions [224].

Fourier-transform infrared spectroscopy (FTIR) measures the absorption of infrared radiation in tissue samples, thus providing information about the molecular and structural tissues composition. FTIR images revealed a decreased collagen maturity and an increased carbonate-to-phosphate ratio at the vertebrae endplate of *Chihuahua* zebrafish mutant compared to WT [61].

#### Mechanical Properties Analysis

The availability of different zebrafish models for skeletal diseases required the down-scaling of specific techniques to investigate biomechanical properties of bone as well as of other tissues. Nowadays stiffness and elasticity can be evaluated at micro/nanoscale level by force spectroscopy methods such as nanoindentation and atomic force microscopy (AFM). Nanoindentation applies higher magnitude force compared to AFM and thus it is preferred for analyzing hard materials, such as skeletal tissues [226].

Nanoindentation was indeed useful to detect alterations of connexons, hemichannels involved in bone growth [227], in the vertebrae of *stöpsel*^*dtl28d*^ [206] zebrafish mutant as well as impaired elastic modulus in the OI zebrafish *Chihuahua* [61]*.* AFM has been used to measure the collagen fiber diameter in zebrafish vertebrae and to calculate the ECM stiffness in regenerating zebrafish myocardium [228].

#### Electrophysiology Assays

Electrophysiology studies can be assessed to investigate heart conduction system. Indeed, electrocardiogram of the *Tg(myh6:cmlc1*^*E17K*^*)* fish revealed a slower sino-atrial activity and an increased heart rate irregularity compared to WT, allowing the characterization of the E17K mutation as causative of atrial fibrillation [229]. Patch clamp analysis can also be performed on zebrafish cardiomyocytes in order to determine ionic currents (i.e. calcium flux) [230]. Furthermore, transmembrane action potential can be evaluated using voltage-sensitive dyes such as di-4-ANEPPS, which intercalates in the cell membranes and emits a red fluorescence in response to variations of membrane potential [231].

#### Skin Functionality Assays

Epidermal permeability or integrity can be investigated by skin functionality assays both in zebrafish larvae and adults. In particular dye penetration assays are often used to determine skin barrier properties, and for instance to evaluate the re-epithelization process during wound healing [138].

## Conclusion

The several already available models for dominant and recessive OI prove the goodness of zebrafish in recapitulating the main OI skeletal features. The easy genetic manipulation paves the way for the rapid generation of all the new OI forms characterized so far and likely of the ones that will be identified in the future. The strong evolutionary conservation of development and structure of tissues/organs such as muscles, heart and skin between teleost and mammals supports the power of zebrafish to investigate also the extraskeletal features of the disease, a clear need for the patients. The possibility to employ zebrafish for high-throughput drug screening taking advantage of the rapid embryonal development, embryo transparency and the availability of specific transgenic lines further strengthen the use of this model in OI and in general in skeletal disease field.
